# Physical and chemical characterizations of a reference e-cigarette used in animal testing

**DOI:** 10.1038/s41598-023-43733-3

**Published:** 2023-10-03

**Authors:** Sébastien Soulet, Léa Constans, Vanille Quinty

**Affiliations:** Ingesciences, 2 Chemin Des Arestrieux, 33610 Cestas, France

**Keywords:** Medical research, Chemistry

## Abstract

A minimal necessary condition for preclinical studies to contribute to risk assessments of e-cigarettes (ECs) is the ability to expose laboratory animals to an appropriate dosage of aerosols. In this study, we examined the fulfilment of this essential consistency condition for the ECX-Joyetech E-Vic Mini (ECX), a piece of computerized exposure equipment manufactured by SCIREQ, which has been employed by numerous in vivo testing. We began by calibrating the customary Evic VTC mini device mod and the 4 coils available, reproducing in the laboratory the operation of the ECX in the power-control and temperature-control modes, using puffing parameters recommended by its documentation. We then conducted the following tests for each coil: (1) verifying whether the generated aerosols satisfy an optimal operational regime, free from overheating, as determined by a linear relation between the mass of vaporized e-liquid vs. supplied power and (2) obtaining the mean yields of aldehydes for each of the tested power settings and coils. The results of these tests show that, under the main conditions used in in vivo testing, the ECX equipment fails to comply with these consistency requirements, especially for coils with low subohm resistance, a shortcoming that can be corrected by applying much larger airflows for these coils. Therefore, the outcomes of preclinical studies using the ECX equipment should be examined with great scepticism and subjected to further testing.

## Introduction

Vaping devices, also known as electronic cigarettes (ECs), are relatively recent consumer products whose aim is to deliver nicotine under safer conditions than tobacco cigarettes. Their design involves a heating element (mainly a wire) that heats and vaporizes a liquid solution (the e-liquid) under boiling temperatures rarely exceeding 300 °C. However, temperature is a thermodynamic state variable that results from heating conditions, which should play a major role in understanding both the chemical releases of emissions and consumer behaviour.

The role of high power settings in open and refillable ECs has been extensively studied in numerous papers undertaking chemical analysis of emissions, with reported levels of aldehydes, carbon monoxide, metals and other compounds significantly higher than in low-powered devices^1–3^. On the grounds of these observations, it is evident that this approach is based on the worst toxicological conditions, with the highest risks for users. A similar approach can be found in many articles using these worst risk exposure conditions in studying the effects of EC emissions on cell cultures^4–6^ and on laboratory animals^7–9^.

However, the power supplied, and more precisely the heat flux, is the key parameter in assessing thermal problems and processes. In the case of vaping products, operational functionality is linked to boiling phenomena^10^. Above specific conditions known as the “critical heat flux”, boiling does not lead to the formation of gas bubbles but to a layer of gas surrounding the wire, whose thickness increases with power, creating a local drying effect.

This drying effect and the resulting pyrolysis of the cotton element in the wick is commonly called a “dry hit”, regarded as a harmful undesirable phenomenon that signals a malfunction caused by a defect in the device design^11,12^. This effect can be initiated even in devices with high manufacturing quality. There is extensive literature on the pyrolysis of cellulose from wood burning^13,14^ and from the food industries, as some of the released chemical compounds (for example, furans) are perceptible and useful as flavours^15^. Therefore, the initiation of a drying process is slightly perceptible at the maximum power settings of EC and becomes increasingly perceptible with increasing power levels^16^.

On the one hand, some recommendations on usage requirements should be provided by the EC manufacturers, but on the other hand, testing conditions should be clearly specified when EC aerosols are laboratory tested for experimental purposes. In two recent extensive reviews, we highlighted this lack of information in numerous studies that looked at the presence of metals, aldehydes, free radicals and carbon monoxide in EC emissions^17,18^. The main problem we found was the testing of high powered subohm EC devices by puffing them with a low airflow rate, led to an excessive presence of toxic byproducts in the emissions, a phenomenon that does not occur when applying high airflow rates to these devices^19,20^.

Similar research as that on emissions needs to be undertaken to review the testing of preclinical effects in biological systems (cell lines and laboratory animals), as these experiments also require appropriate emission generation. Numerous studies have examined preclinical effects from EC emissions using the ECX kit, a commercially available computerized exposure equipment manufactured by SCIREQ (Scireq®, Montreal, QC, Canada)^21^. Unfortunately, SCIREQ does not provide sufficient publicly available information on this tool, and most studies using this equipment only cite the SCIREQ provider and/or only report the device and the coil they used but not the full set of conditions. Another problematic issue is the usage of a device with a custom-made tank (Subtank 7 mL) as a reference device by SCIREQ, without qualifying it, which makes experimental conditions in the different laboratories using the ECX kit likely incompatible with the requirements given by KangerTech, the manufacturer of the original clearomiser.

The aim of the present paper is to reproduce the results obtained by the device defined as a reference by SCIREQ for in vivo studies. For this purpose, we had to search for the same clearomiser and the same coils, which are no longer manufactured. Unfortunately, we were unsuccessful in finding any remaining devices, so we used the Toptank instead, whose design is very similar to the Subtank used by the ECX kit and is equipped with the same coils (same size and more importantly, close air inflow opening). To characterize this device, we performed three steps: (1) off-power electric calibration of the box used (JoyeTech EVIC VTC Mini); (2) determination of the limits of optimal operational functionality by testing the mass of e-liquid vaporized; and (3) quantification of aldehyde yields to highlight the consequences of overheating and to qualify the relevance of the experiments.

## Materials and methods

Since the risk of overheating depends on the supplied power, the first task was the electric calibration of the EVIC VTC mini. This task is very important, as the batteries are commercial products not intented for laboratory testing.

### General and technical information of the four coils used with the Toptank

The information provided by the e-cig manufacturer is reported in Table 1.

**Table 1:** Information marketed with each coil.

Reference	Resistance advertised (Ω)	Alloy	Power range (W)
OCC 0.15 Ω	0.15	Nickel	15–50
SSOCC 0.5 Ω	0.50	SUS316L	15–60
SSOCC 1.2 Ω	1.20	Nichrome	7–15
SSOCC 1.5 Ω	1.50	Nichrome	12–25

However, the information available on the internet highlights that KangerTech manufactured two versions of the same coils, denoted the SSOCC and SSOCC V2 coils. A request for clarification was sent on the 25th of January 2023 following the procedure specified by the KangerTech website (https://www.kangertech.com/contact/), without receiving any response (as of the 27th of April). Therefore, the accuracy and scope of our approach is necessarily limited by the scarcity of the information provided by authors using the ECX kit, the available information from SCIREQ and by the confusing information from KangerTech.

The Subtank and Toptank are devices with large air inflow. Based on the method developed in a previous work^22^, we completed the technical information with the heating surface and the air resistance, allowing clearer identification of the intended usage (Table 2).

**Table 2:** Technical information determined following the methods from^22^.

Reference	Resistance measured (Ω)	Alloy	Length (mm)	Diameter (mm)	Surface (mm^2^)	Air resistance (Pa^0.5^ min L^−1^)
OCC 0.15 Ω	0.20	Nickel	150	0.202	95	1.76
SSOCC 0.5 Ω	0.71	SUS316L	43	0.255	35	1.72
SSOCC 1.2Ω	1.48	Nichrome	54	0.227	39	3.45
SSOCC 1.5Ω	1.81	Nichrome	27	0.147	12	6.07

Based on previous observations, the low air resistance would foster inhalations with high airflow rates rather than low ones as defined in the ISO 20768 puffing regimen^23^.

### Electrical calibration of the EVIC VTC power control and temperature control modes

The battery was screwed onto a bracket connected to a multimeter (RS PRO, IDM 505), allowing recording of the value of the current and the supplied voltage while the battery was manually activated for 10 s. This bracket was fixed at an adjustable resistance of 4.7 Ω max 250 W. The calibration started at 10 W and was increased by 5 W increments until reaching 75 W or lower if the box (devices containing the accumulator and regulating the power supplied) displayed an error message. The calibration was performed by fixing an equivalent resistance, with a value (0.16 Ω, 0.51 Ω, 1.22 Ω, 1.52 Ω) as close as possible to the ones advertised (0.15 Ω, 0.5 Ω, 1.2 Ω, 1.5 Ω).

Then, a similar experiment was performed using the temperature control mode on the OCC 0.15Ω at temperature settings from 100 to 315 °C with a step of 25 °C. The temperature (denoted T) was evaluated using the evolution of the resistance value (denoted R(T)) following the linearization of the Callendar-Van Dusen equation^24^:

1 $$\mathrm{R}\left(\mathrm{T}\right)={\mathrm{R}}_{0}\cdot \left[1+\mathrm{\alpha }\cdot (\mathrm{T}-{\mathrm{T}}_{0})\right]$$

where T_0_ is the ambient temperature, R_0_ is the initial resistance value measured at T_0_ and α is the temperature-resistance coefficient, determined to be 0.0057 °C^−1^ (see Supplementary Annex S2).

### Determination of the functional limits of the four coils used with the Toptank

In operation, the characterization was focused on the determination of the minimum and maximum power. Each coil was deliberately tested on a power range above the manufacturer’s recommended range. The mass of e-liquid vaporized was measured as the mass difference of the e-liquid filled atomizer before and after the experiment^25^ using a calibrated Mettler AT261 DeltaRange laboratory scale (range from 1 mg to 205 g, with a precision of 0.1 mg).

Each experiment was performed in triplicate at each power setting by applying a puffing regime consistent with the device requirements. Two puffing regimes were used to account for low-/high-power devices. The one with the low airflow rate was defined by the ISO 20768 standard (3 s puff duration, 1.1 L/min), whereas the second was designed and explained in a previous paper (3 s puff duration, 10 L/min)^19^.

The power was supplied by a U-SAV vaping machine designed with efficient automation to regulate the voltage in real time^26^. Table 3 summarizes the tested conditions.

**Table 3:** List of the conditions tested.

Reference	Power tested	Vaping regime	Liquid
OCC 0.15 Ω	10–50 W by step of 4 W	3 s puff, 30 s period, 1.1 L/min, 20 puffs 3 s puff, 30 s period, 10 L/min, 20 puffs	50/50 PG/VG 2% nicotine
SSOCC 0.5 Ω	10–70 W by step of 4 W	3 s puff, 30 s period, 1.1 L/min, 20 puffs 3 s puff, 30 s period, 10 L/min, 20 puffs	50/50 PG/VG 2% nicotine
SSOCC 1.2 Ω	5–29 W by step of 3 W	3 s puff, 30 s period, 1.1 L/min, 20 puffs	70/30 PG/VG 2% nicotine
SSOCC 1.5 Ω	5–32 W by step of 3 W	3 s puff, 30 s period, 1.1 L/min, 20 puffs	70/30 PG/VG 2% nicotine

### Quantification of the main aldehydes released by the four coils used with the Toptank

Progressive overheating conditions were observable from the released aldehydes. For that purpose, the generation of emissions was also performed by coupling the vaping machine to an mpinge filled with 20 mL of DNPH-solution. This solution was prepared in a higher volume and consisted of 1.1 g of 2,4-dinitrophénylhydrazine (CAS: 119-26-6, > 97%) in 500 mL of methanol (CAS: 67-55-1, HPLC ULTRA gradient) and finally completed with 10 mL of formic acid (CAS: 64-18-6, < 98%).

When the experiment ended, 500 µL of the solution in the mpinge was collected and injected into a UHPLC chromatograph (ACQUITY UPLC H-Class PLUS System waters®) equipped with a RAPTOR C18 Column 2.7 µm-4.6 × 150 mm (Restek®) coupled with a UV detector (PDA waters®) and a mass spectrometer (QDA waters®). The method developed used an initial solution of 500 µL of a standard of formaldehyde-2,4-DNPH (100 µg/mL in acetonitrile), 50 µL of a standard of acrolein-2,4-DNPH (1000 µg/mL in acetonitrile) and the same volume of a standard of acetaldehyde-2,4-DNPH (100 µg/mL in acetonitrile) brought to a final volume of 10 mL with ethanol. Two ranges of calibration were used: a lower range quantifying concentrations from 0.1 to 0.5 µg/mL and a higher range from 0.5 to 2 µg/mL standard solutions. The sample required was made from the initial solution diluted in methanol. When the sample collected from the mpinge provided results outside the higher range, the mpinge sample was diluted in methanol.

## Results

The results are presented in a progressive way, allowing a step-by-step understanding of the conditions applied using the ECX kit equipment for EC emissions testing.

### Electrical calibration of the EVIC VTC

#### Evaluation of the electric efficiency in power control and temperature control modes

The first step was the characterization of the EVIC VTC as an electric source for energy supply.

**Figure 1:**
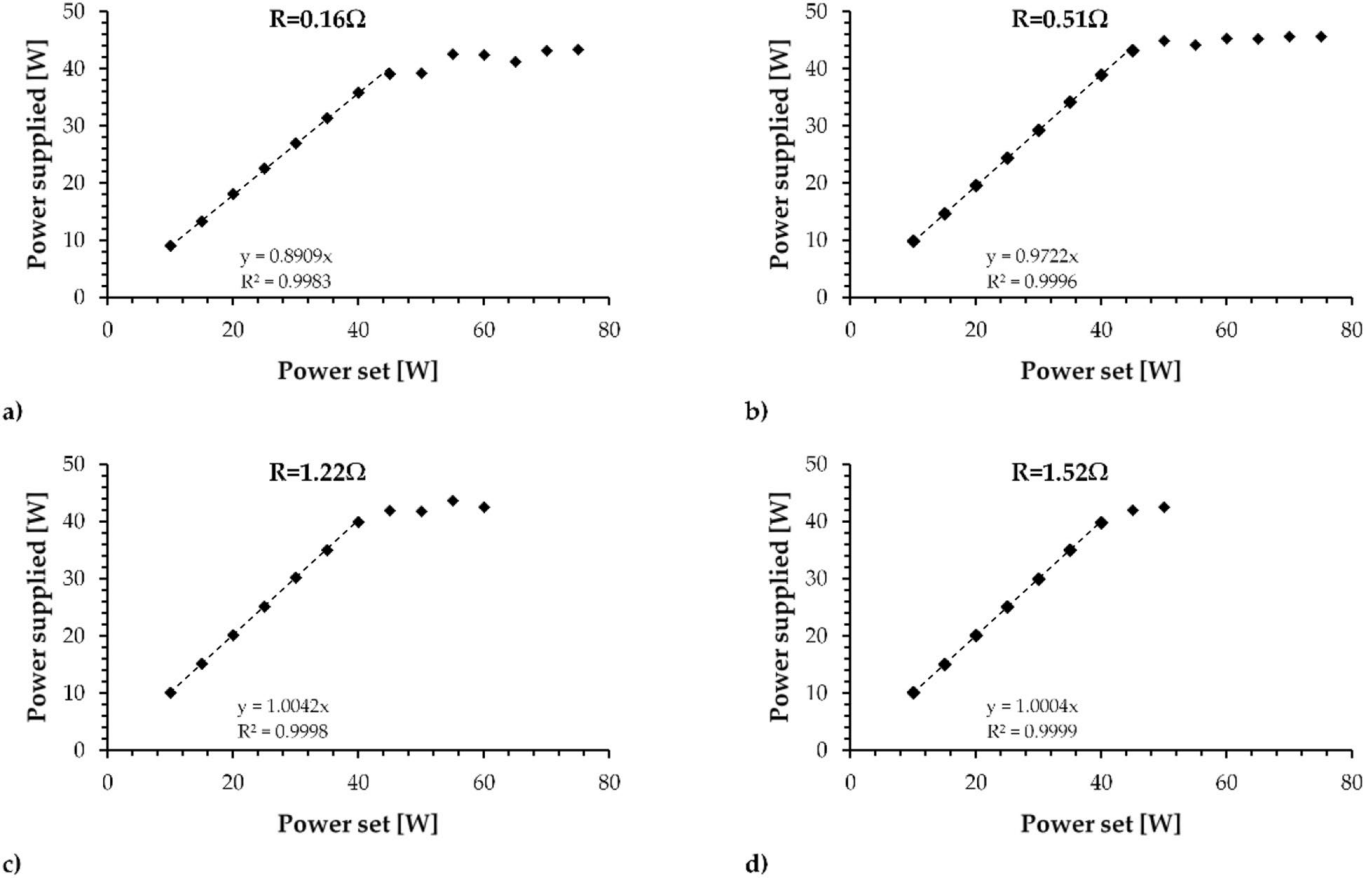
Electric qualification of the EVIC VTC box tested with the adjustable resistance fixed at (**a**) 0.16 Ω, (**b**) 0.51 Ω, (**c**) 1.22 Ω and (**d**) 1.52 Ω.

Figure 1 shows the electrical calibration of the box tested with the adjustable resistance fixed at 0.16 Ω, 0.51 Ω, 1.22 Ω and 1.52 Ω.

Based on the calibration curve, the EVIC VTC has an electric efficiency of 89% below 45 W set using 0.16 Ω and 97.2% below 45 W set using 0.51 Ω, reaching 100% on the 1.22 Ω and 1.52 Ω coils. In both tests, the supplied power of the box reached a limit of approximately 43 W. Based on these observations, the following results are reported following the supplied power to prevent misunderstandings caused by the difference between the power set by the device and the supplied power.

#### Evaluation of the electric efficiency in temperature control mode

**Figure 2:**
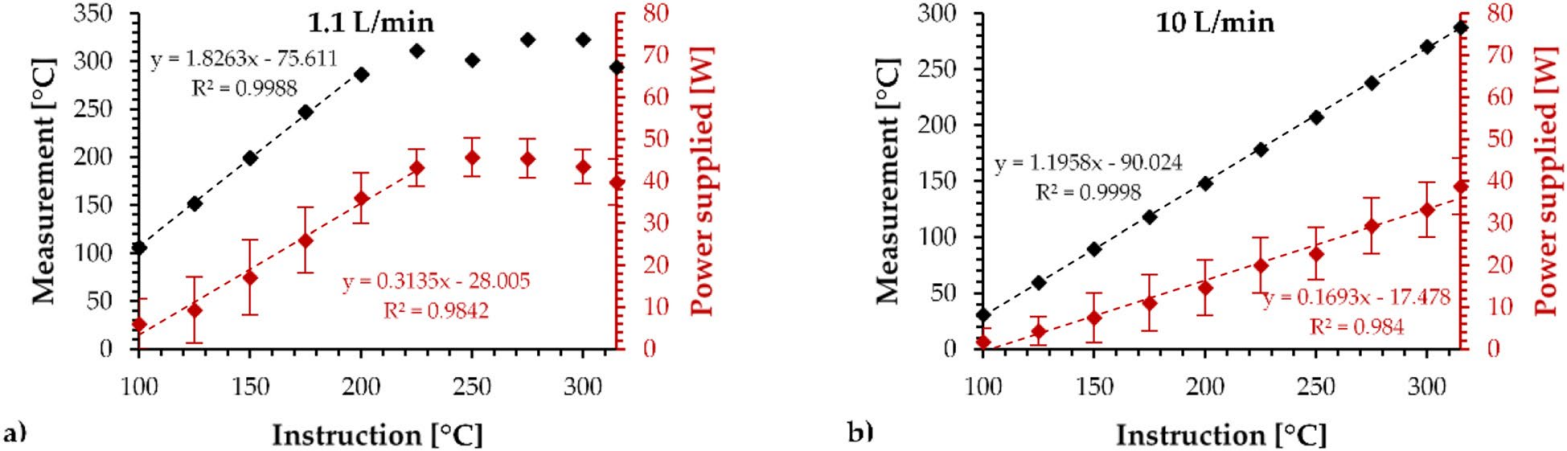
Temperature reached at the end of the 10 s when the EVIC-VTC mini was used in temperature control mode applying (**a**) 1.1 L/min and (**b**) 10 L/min on the OCC 0.15 Ω.

Figure 2 provides the calibration results using the temperature control mode with the two airflow rates on the OCC 0.15 Ω.

As shown by the 1.1 L/min graph, the estimated temperature reached a value in 10 s that followed a linear trend with the set temperature below 200 °C. Above 200 °C, the estimated temperature remained a constant value. Based on the average supplied power, the regulation was blocked by the maximum specified by the box.

As shown by the 10 L/min graph, the temperature reached a value that clearly followed a linear trend according to the set temperature, which was observable over the full tested range. Then, the supplied power values during the tests were lower than those supplied using 1.1 L/min. This means that the temperature regulation was more efficient (wider range) and more accurate (slope closer to 1), clearly outperforming the temperature regulation using a low airflow rate.

### Determination of the functional limits of the four coils used with the Toptank

Once these experiments were completed, we displayed the function curves of each coil under the specific vaping regime. All curves showed the same characteristics, with the minimum and maximum power values at the extremes lying outside the linear region that defines the optimal functionality regime.

**Figure 3:**
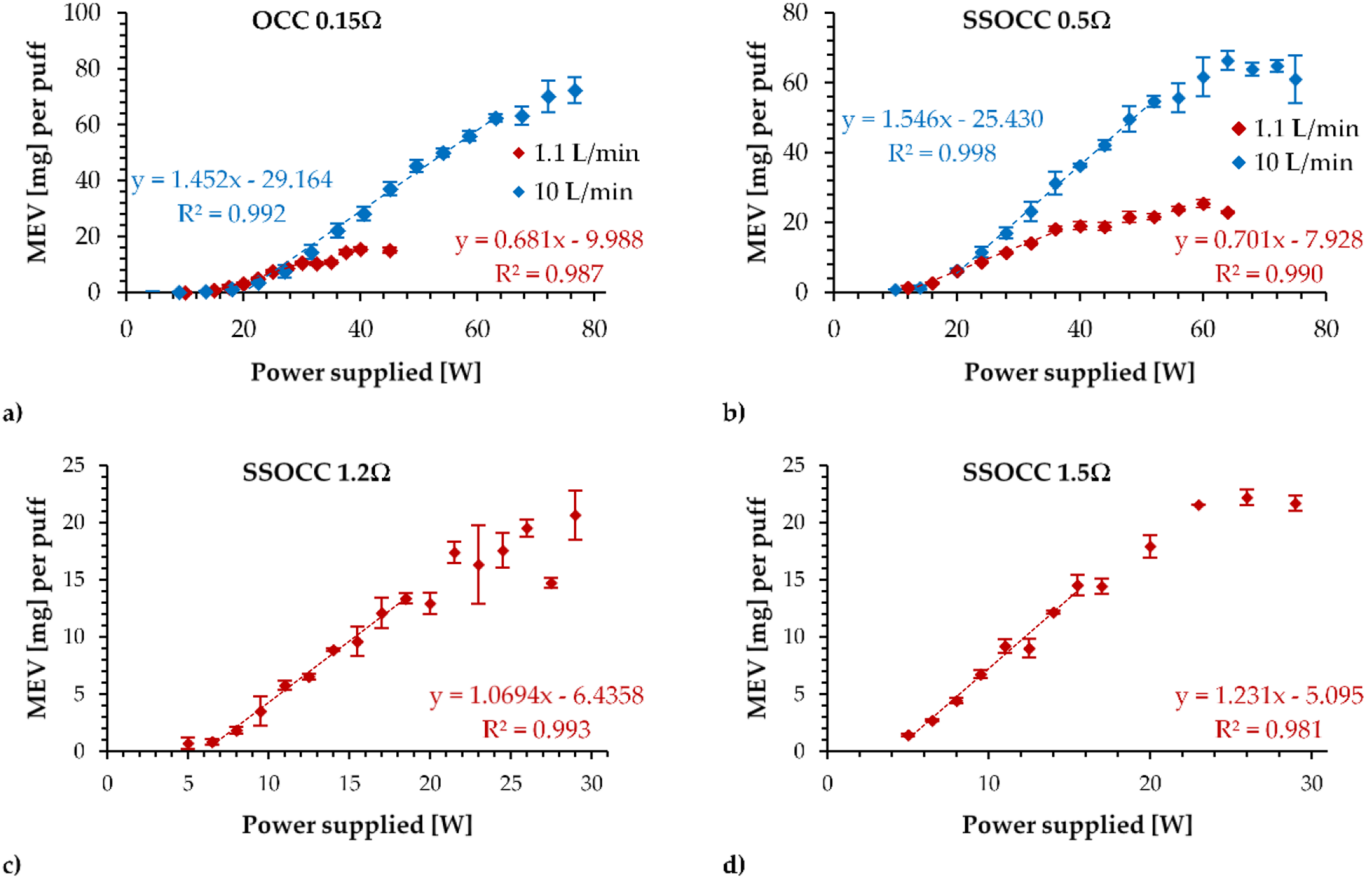
Operational functionality curves of (**a**) OCC 0.15 Ω, (**b**) SSOCC 0.5 Ω, (**c**) SSOCC 1.2 Ω and (**d**) SSOCC 1.5 Ω coils using 1.1L/min (in red) and 10L/min (in blue) airflow rates (only for low resistances).

The operational functionality curves obtained from the generation of emissions are depicted in Fig. 3.

The operational functionality curves of the OCC 0.15 Ω were very different when using low and high airflow rates. With a low airflow rate, the optimal regime was restricted to a narrower range between 14.7 W and 29.7 W, while the range extended between 20.1 W and 63.4 W when applying a high airflow rate.

The same observation held for the SSOCC 0.5 Ω operational functionality curves. With a 1.1 L/min, the range for optimal conditions was between 11.3 W and 38.5 W, whereas the range was between 16.6 W and 52.5 W at 10 L/min.

Finally, the optimal power range for the SSOCC 1.2Ω was between 5.9 W and 17.6 W when applying the standard vaping regime, and that of the SSOCC 1.5Ω was between 3.7 W and 19.8 W.

### Quantification of the main aldehydes released by the four coils used with the Toptank

**Figure 4:**
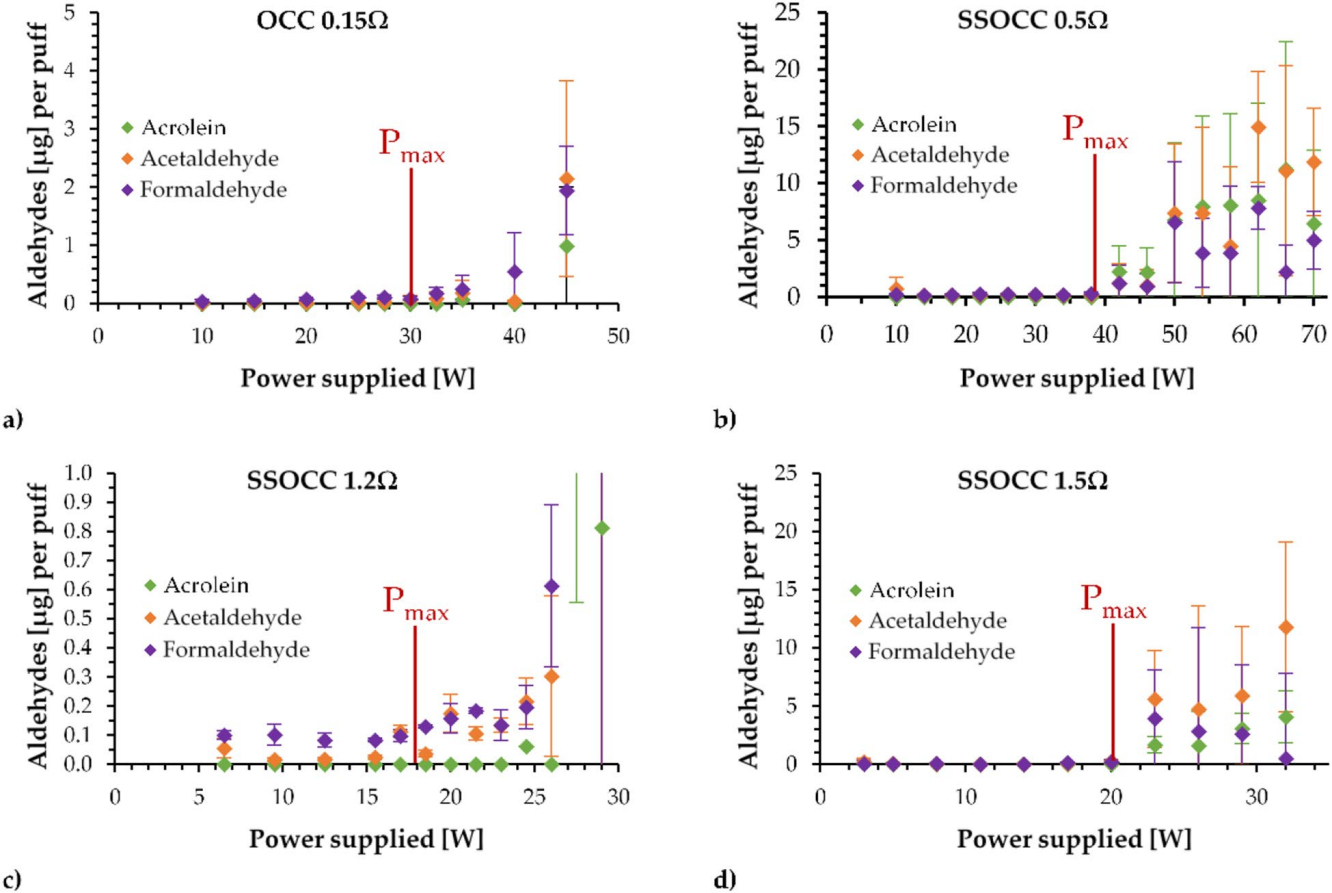
Aldehydes released using the (**a**) OCC 0.15 Ω, (**b**) SSOCC 0.5 Ω, (**c**) SSOCC 1.2 Ω and (**d**) SSOCC 1.5 Ω applying a low air flow rate (P_max_ = maximal power). Due to scale issue with the SSOCC 1.2Ω, the results at 27.5W and 29W have been hidden but they reach values close 10 µg per puff.

Figure 4 provides the quantification of the yields of the targeted aldehydes (Acrolein, Acetaldehyde and Formaldehyde) as functions of the supplied power for the four coils. The maximal functionality powers determined in the previous section are shown as markers of the onset of overheating conditions.

For each device, experimenting above the maximal power leads to a progressive increase in the aldehydes released. Below this specific power, their quantities remain below l µg generated per puff.

## Discussions

Our results lead to the discussion of many critical points on how the characterization of emission generation influences the physical and chemical properties of an aerosol, which are relevant factors in toxicological evaluations. Therefore, the aerosol generation process is a highly important and necessary condition for these experiments to be useful for public health assessments on the safety of ECs.

### Thermal science of electronic cigarettes and toxicological assessment

Recently, we published a paper dealing with the thermal engineering of electronic cigarettes. We explained how boiling regimes influence the heat transfers between the wire and the e-liquid and highlight, using correlations, how specific heat flux of boiling regimes lead to power limits for e-cigarettes^27^. The results of the present characterisation on SSOCC coils are also linked to boiling regimes. However, the aldehydes quantified support the implication of boiling in toxicological assessments of vaping use. Below the maximal power, the aldehydes remains at low but not-negligible values while above this notable power, the quantities increase significantly. This important issue results from overheating conditions induced by film boiling where a wire is surround by the e-liquid in gas phase and no more in liquid phase. Below the maximal power, vaporisation occurs under thermodynamic equilibrium and the gas is released in the airflow^28^. Under film boiling, the liquid is no more in contact with the wire and the gas resulting from the e-liquid vaporisation surrounds the wire. These conditions allow continuing the heating of this gas above the boiling temperature (called overheating of the e-liquid). Following Arrhenius relation describing chemical reaction rate^29,30^, it is obvious that suppling power above the maximal power exponentially increase the aldehydes released as it is observable with the SSOCC coils.

### Worst conditions of use and high power

A large share of currently available vaping devices allow adjustment of the coil resistance (though not the battery output) and often the supplied power with a power range recommended by manufacturers. The basis behind this power range is unclear, but for most of the range of use in consumer products, we can assume that the lower end values mark a sort of safety default, while the upper end signals the onset of a characteristic excess. In addition to power settings, some vaping devices also allow adjustable e-liquid composition, nicotine levels and airflow rate. Food products are a useful analogy, where cooking is an adjustable multiparameter experiment, and the training of the cook is based on learning to adjust these parameters to reach an ideal dish based on the criteria of its organoleptic characteristics (taste).

The optimal operational functionality of vaping devices can be experimentally determined by a regime in which the increase in supplied power produces a linear increase in the mass of e-liquid vaporized, which involves a consistent proportional increase in the released quantities of nicotine, flavourings and aldehydes. However, the upper end of this power range marks the onset of an exponential enhancement of chemical reactions that generate aldehyde production linked to the onset of overheating conditions and pyrolytic degradation of the cellulose in the wick. The literature on organoleptic perceptions during vaping is sparse, but a few papers have investigated perceptions when a dry hit occurs^16,31^. The pyrolysis of cellulose material releases flavouring compounds that are well known in the food industry for their burnt taste. Therefore, it is quite plausible to assume that power above the optimal functionality should lead to an alteration in the normal perception of e-liquid flavours, likely in the form of additional unexpected flavours. In the low-power extreme, there is practically no perception, as the mass of e-liquid vaporized is close to nil.

On the grounds of the experimental evidence we have obtained and previous discussions, we can assume that the power range recommended by the manufacturer should be at least partly based on perception criteria based on ranges in which vaping has been observed to be pleasant for testing volunteers, a range that should be contained within the range of the optimal functionality regime. In other words, it is possible to conceive a qualitative measurement of the pleasant sensation during vaping as a sort of Gaussian distribution curve, with the tails around the minimum and maximum optimal powers and approximately centred on the best reported taste. While taste can be subjective and subject to individual variation, this might explain why the manufacturer recommendations for some coils lie in a relatively narrow band within a wider range of power allowed by the EC device. All this raises a serious question regarding the best and worst possible conditions for toxicological assessments.

Evidently, the worst toxicological conditions are associated with either the highest powers above the recommended ranges or the combination of the optimal regime with a low airflow. Fixing such settings in a box mod used for testing is equivalent to the assumption that a user is forced to vape under such settings, unable to adjust the parameters guided by organoleptic perceptions, so if overheating conditions appear, the user will simply continue vaping. This behaviour can certainly occur in vaping machines but cannot justifiably be expected of human users guided by a pleasant experience during a recreational activity. The worst toxicological conditions associated with overheating might occur in real life during one or a few puffs, but this is a temporary event whose continuation users will not tolerate; hence, such an unpleasant short-term event does not characterize a pattern of daily consumption.

As in the case of other products, a realistic toxicological assessment must be based on the observed dosage (daily number of puffs or daily quantity of e-liquid consumed) but must also consider the wattage range and puffing parameters that best represent user behaviour.

### Relevancy of the experiments and normal use

The literature on consumer usage conditions is scarce, but one paper provided statistical information on how two of the four coils tested (0.5 Ω and 1.5 Ω) are used by experienced vapers^32^. The study enrolled 32 vapers, with the authors providing a device (with the coils) and a specific e-liquid. The powers were set manually by users at 40.5 W and 13.5 W for the 0.5 Ω and 1.5 Ω coils, respectively. The “manual manipulation” step is equivalent to the usage learning described above. Their results highlight an average puff volume (and puff duration) of approximately 480–520 mL (3–4 s) with an e-liquid containing 4 mg/mL and 360–380 mL (2.3–3.3 s) with an e-liquid containing 8 mg/mL.

Despite the small sample of vapers enrolled, it is clear that for experienced users, the 0.5 Ω coil is not intended for puffing conditions close to the ISO 20768 or CORESTA recommended method 81. The reason behind this result is that the airflow rate plays a major role in the functioning of the device, as it increases the heat exchange from the wire to the surrounding air that forces its cooling. A very low and insufficient airflow makes this cooling far less efficient and promotes early overheating conditions.

Then, setting 40.5 W in the KBox mini (box provided by the authors) leads to a supplied power of 36.9 W (Efficiency = 91%, see Supplementary information S1). Under these conditions, experimenters are within the optimal vaping functionality regime of the coil, leading to normal conditions of use, whereas applying a significant restriction of the airflow (by closing the air inflow or by lip restrictions) would lead to overheating conditions. This consideration is very important, as it changes all the results of an experiment, a mistake that often occurs in experiments aimed at quantifying the compounds in EC emissions.

The development of a testing standard is a long process that requires data to develop and a consensus for its publication and implementation. The ISO 20768 standard was published in 2018, at a time when the literature used for its development was mainly based on puff topography experiments on the first EC “ciga-like” products available in the market. As the market has significantly changed from these products to open systems, from high to low air resistance and from low- to high-power devices and (more recently) back to more advanced low-power ones, this standard is now obsolete or unusable for many (or most) devices. This lack of an updated protocol is leading to the development of a new standard, but only a few articles have considered the necessary adaptations to test high-powered devices used with direct lung inhalation^19,20^. Since this standard is not yet available or published, most researchers continue designing their studies using the old standard or with ad hoc protocols. Therefore, it is the responsibility of providers of laboratory equipment to take into consideration the different normal and abnormal conditions of use.

### Scireq equipment

A single technical point provides a warning sign on the outcomes of experiments using the ECX kit equipment for the generation of emissions. The video providing the description and explanation of the equipment, linked to the publication of the protocol, depicts a homemade tank designed to replace the original Pyrex Subtank, filled with 10 mL of e-liquid that does not cover the full coil part attached to the mod box. The equipment is kept in a horizontal position during the whole experiment, with the e-liquid level decreasing even farther below the coil area. It is important to note that the initial e-liquid is colourless, whereas it becomes brown at the end of the experiment.

There is one main reason for this browning of the e-liquid: pyrolysis of the cotton element. A dry hit is commonly understood as a fast pyrolysis of cotton, but in reality, the pyrolysis of an organic structure such as cotton (i.e., mainly in cellulose) is a gradual process. This gradual process reaches a spontaneous point under abnormal conditions. There is extensive literature on cellulose pyrolysis, and one of the first steps of this degradation leads to the release of bio-oil beginning at 300 °C^13^. This oil is a dark-brown liquid that becomes increasingly lighter as it dilutes, changing colour from light brown to slightly yellow^33^.

The horizontal position and the large width of the homemade tank necessary lead to one e-liquid inlet being partially or totally in contact with air. Under these conditions, the wetting of the cotton might take place through the second e-liquid inlet immersed in the e-liquid. These unusual conditions are likely to promote the occurrence of a dry hit.

### Implications for animal testing

The Animal Welfare Act (AWA) regulates the use of animals for laboratory testing. Following the AWA requirements, the Public Health Service Policy (PHS) provides principles for the utilization and care of vertebrate animals used in testing, research, and training. Principle II should be cited, as the results of this paper and the discussions raise question about the ethics behind some papers already published.

*“Procedures involving animals should be designed and performed with due consideration of their relevance to human or animal health, the advancement of knowledge, or the good of society.”*

Principle II, Public Health Service Policy (PHS) on

Humane Care and Use of Laboratory Animals

As discussed in the previous points, the conditions used for the generation of emissions are inconsistent with realistic use of the devices, implying that compounds generated by the overheating conditions are very likely to exacerbate the quality of the aerosols used to expose laboratory animals. Therefore, the health and physiological outcomes observed in mice, rats or hamsters exposed to aerosols generated under abnormal conditions are questionably relevant for users and therefore stand in opposition to principle II. The Organization for Economic Co-operation and Development (OCDE) provides guidance on inhalation toxicity studies, and point 62 of document 39 should also be cited, as it provides interesting guidelines:

*“The feasibility of generating a targeted test atmosphere should be determined in a test without animals. […]. Tests are mandatory […] to prevent useless animal exposures. Each test chemical may pose unique physical challenges and/or require vehicle systems to generate and characterize the test atmosphere.”*

Document 39, point 62, Organisation for

Economic Co-operation and Development

The OCDE recommends carrying out preclinical studies with prior chemical analysis to avoid useless animal exposures. To apply these recommendations to preclinical studies using the ECX equipment, we recommend providing the following:

- a clear description and characterization of the tested device;
- identification of normal conditions of use;
- quantification of the exposure to concentrations of hazardous compounds.

## Conclusion

In this article, we have provided a step-by-step characterization of the ECX equipment manufactured by SCIREQ (Scireq®, Montreal, QC, Canada). The measurements of the electrical efficiency of the device in both power and temperature control modes highlight that the regulation of these parameters is not rigorous or accurate and should be calibrated for laboratory purposes. The determination of the maximum values of a functionality power range marks a starting point that can be associated with unpleasant perception induced by the chemical degradation of the cotton element in the wick. Finally, we obtained a chemical quantification of aldehyde yields that provides useful information on power ranges for a realistic toxicological evaluation.

The JoyeTech EVIC mini used in the ECX kit activates the mod box at a fixed default value of 70 W, even in the temperature control mode. As a consequence, the device necessarily supplies 70 W in the first puffs, adjusting power levels afterwards to approximately 40 W. Under these conditions, experiments applying a low airflow rate and a constant horizontal position of the device contribute to inconsistent results based on aerosols that are not representative of aerosols inhaled by users of this type of device.

A minimally necessary condition for animal models to provide useful clues to assess the effects of EC aerosol exposure in human subjects is the usage of aerosols generated without overheating under conditions that best translate between normal human usage and animal scale of exposure. We have found at least 15 papers published between 2020 and 2023 that examined EC aerosol exposure in animal models (adult mice, rats, hamsters and pregnant mice to observe adverse effects in offspring). All used the ECX equipment, with the same box device and coils that we have tested in the present article, thus generating aerosols under the inconsistent overheating conditions that we have described, which are unrepresentative of the normal usage of ECs. These studies do not comply with the basic relevant criteria that we have extensively discussed; hence, their results are questionable and must be taken with scepticism.

Research on vaping products has focused on chemical or biological testing without sufficient knowledge on the functioning of the devices used in their experiments, ignoring the physical processes behind the aerosol generation process. Most researchers conduct their experiments without awareness and/or willingness to investigate (and incorporate into the study design) a wide variety of parameter modifications that characterize real usage patterns. Most researchers also downplay the importance of consumer satisfaction based on sensorial perception of the aerosol and flavours, all of which support their acceptability for smoking cessation. Unfortunately, a significant share of research on ECs is more concerned with detecting and extrapolating harms than with understanding how the devices operate and why they have become accepted and used as less harmful nicotine delivery systems by millions of smokers and ex-smokers. We hope that the present article can convey to researchers on emission and preclinical studies of ECs to base their experimental work on the best possible approximation to realistic usage and understanding of the physical processes under their optimal operation.

### Supplementary Information

Supplementary Information.

## Data Availability

The data presented in this study are available in this article.
