# Author Correction: Physical and chemical characterizations of a reference e-cigarette used in animal testing

**DOI:** 10.1038/s41598-023-44863-4

**Published:** 2023-10-17

**Authors:** Sébastien Soulet, Léa Constans, Vanille Quinty

**Affiliations:** Ingesciences, 2 Chemin Des Arestrieux, 33610 Cestas, France

Correction to: *Scientific Reports* 10.1038/s41598-023-43733-3, published online 03 October 2023

The original version of this Article contained an error in the name of author Léa Constans, which was incorrectly given as Léa Constant.

The original Article has been corrected.

