# Supplementary material for: Physical and chemical characterizations of a reference e-cigarette used in animal testing
Source: Sci Rep. 2023 Oct 3;13:16624. doi: 10.1038/s41598-023-43733-3 (PMC10547745; doi:10.1038/s41598-023-43733-3)
Supplement: Supplementary file 1 — Supplementary Information. [file 41598_2023_43733_MOESM1_ESM.docx]

# Annex 1: Example of EVIC VTC mini temperature regulation at 250°C set

An example at 250°C set (Figure 1), 46W were supplied using 1.1L/min whereas 23W were supplied with the 10L/min (in average).

Figure 1 - Example of the power supplied curves at 250°C set using the 1.1 L/min and 10L/min airflow rates.

As observable, there is an initial shoot leading to 46W supplied. Temperature control requires an initial instruction that will be supplied before the regulation is activated and as observed in Figure 2.d, the 70W set for regulation leads to this 46 W supplied in reality.

# Annex 2: Calibration of the temperature coefficient of resistivity

The temperature coefficient of resistivity is determined by carrying hot wire testing consisting in measuring the temperature reached by a wire of 8cm-length and 32Ga-diameter in Nickel when the voltage is progressively increased (from 0.1V to 1.5V by step of 0.1V). The results are illustrated in Figure 2.

Figure 2 - Evolution of the resistance according to the temperature measured through a hot wire experiment with a K-probe.

From Figure 2, the slope expressing the α is determined at 0.0057 °C^-1^. This value is then used to convert the evolution of OCC 0.15Ω coil in an estimated temperature.

# Annex 3: Calibration of the coefficient of temperature resistance

Figure 3 provides the electric calibration of the KBOX using the adjustable resistance fixed at 0.5Ω and 1.5Ω.

|  |  |
| --- | --- |

Figure 3 - Electric qualification of the KBOX tested with an adjustable resistance fixed at a) 0.5Ω and b) 1.5Ω.

The electric efficiency of the KBOX is respectively 84.03% and 96.06% with the resistance fixed at 0.5Ω and 1.5Ω.
